# Circulating Nucleic Acids as Novel Biomarkers for Pancreatic Ductal Adenocarcinoma

**DOI:** 10.3390/cancers14082027

**Published:** 2022-04-17

**Authors:** Ryan McGowan, Áine Sally, Anthony McCabe, Brian Michael Moran, Karen Finn

**Affiliations:** 1 Department of Life Sciences, School of Science, Atlantic Technological University Sligo, Ash Lane, Ballytivnan, F91 YW50 Sligo, Ireland; ryan.mcgowan@mail.itsligo.ie (R.M.); mccabe.tony@itsligo.ie (A.M.); 2 Department of Analytical, Biopharmaceutical and Medical Sciences, School of Science and Computing, Atlantic Technological University Galway City, Dublin Road, H91 T8NW Galway, Ireland; aine.sally@research.gmit.ie (Á.S.); brian.moran@gmit.ie (B.M.M.)

**Keywords:** PDAC, biomarkers, miRNA, personalized diagnostics, liquid biopsy

## Abstract

**Simple Summary:**

Pancreatic ductal adenocarcinoma (PDAC) is a particularly aggressive and deadly cancer, primarily due to failure to identify early-stage disease. PDAC is often diagnosed at a late stage due to nonspecific symptoms, and a distinct lack of reliable biomarkers for timely diagnosis. Current PDAC biomarkers are inadequate for the monitoring of a patient’s response to treatment. The aim of this review is to highlight the potential use of circulating nucleic acid-based biomarkers, along with technology facilitating their detection, in liquid biopsies. These biomarkers primarily focus on the detection of PDAC-specific genetic mutations, both freely circulating and contained within exosomes.

**Abstract:**

Despite considerable advancements in the clinical management of PDAC it remains a significant cause of mortality. PDAC is often diagnosed at advanced stages due to vague symptoms associated with early-stage disease and a lack of reliable diagnostic biomarkers. Late diagnosis results in a high proportion of cases being ineligible for surgical resection, the only potentially curative therapy for PDAC. Furthermore, a lack of prognostic biomarkers impedes clinician’s ability to properly assess the efficacy of therapeutic interventions. Advances in our ability to detect circulating nucleic acids allows for the advent of novel biomarkers for PDAC. Tumor derived circulating and exosomal nucleic acids allow for the detection of PDAC-specific mutations through a non-invasive blood sample. Such biomarkers could expand upon the currently limited repertoire of tests available. This review outlines recent developments in the use of molecular techniques for the detection of these nucleic acids and their potential roles, alongside current techniques, in the diagnosis, prognosis and therapeutic governance of PDAC.

## 1. Introduction

Pancreatic ductal adenocarcinoma (PDAC) is currently the seventh leading cause of cancer-related death in the world [1]. It is projected to become the second most common cause of cancer-related death by 2030 [2]. The significant mortality rate associated with PDAC is largely due to an inability to detect early stage PDAC; currently only 15–20% of patients have operable tumors at the time of diagnosis. Despite significant improvements in therapeutic options available for PDAC, patient outcomes remain unsatisfactory, with the 5-year survival rate at only 9% [1,3]. Additionally, many patients experience prompt disease recurrence post-surgery [4]. This failure of treatment is caused, in part, by acquired chemoresistance [5]. Chemoresistance in PDAC involves a multitude of factors such as altered gene expression (e.g., *KRAS*, *CCND1*, and *BCL2*) and characteristics including enhanced epithelial–mesenchymal transition (EMT). Another hallmark of PDAC pathophysiology is the dense desmoplastic tumor microenvironment (TME) that consists of stromal and immune regulatory cells. The effects of the TME on key tumorigenic mechanisms such as malignancy, disease progression and drug resistance are caused by the excessive synthesis of extracellular matrix (through key components such as fibronectin and hyaluronic acid), and also infiltration of pro- and anti-inflammatory cells, such as tumor-associated macrophages and T-lymphocytes [6,7].

**Figure 1:**
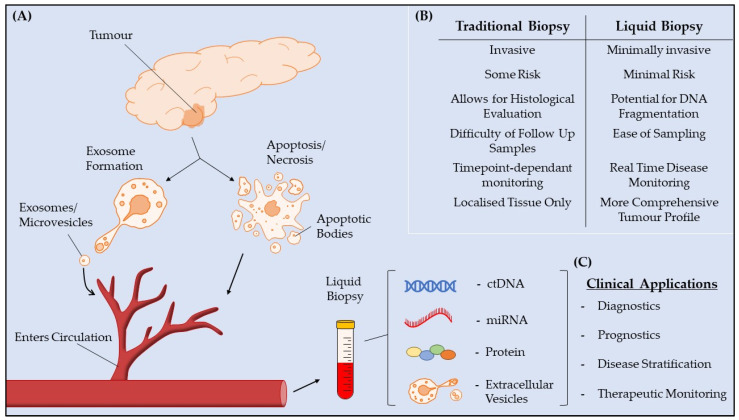
Liquid biopsies for PDAC. (**A**) Mechanisms through which various tumor biomarkers enter circulation. Extracellular vesicles, such as exosomes, microvesicles, and apoptotic bodies bud off from tumor cells and enter circulation. Circulating tumor (ct) DNA, miRNA and proteins are secreted from tumor cells or released through cellular apoptosis and necrosis. (**B**) Comparison between liquid and traditional biopsies. (**C**) Potential clinical applications of liquid biopsy-based biomarkers in PDAC.

MicroRNAs (miRNAs) are small (18–22 nt), endogenous non-coding RNAs that regulate protein output through post-transcriptional modulation [8]. miRNAs alter mRNA expression primarily through binding to the 3′-UTR of their targets resulting in the inhibition of translation or promoting mRNA degradation [9]. Aberrant expression of miRNAs arises in malignant cells due to mutations in miRNA-encoding genes, epigenetic mechanisms and dysfunctional miRNA processing [10]. miRNAs are associated with various tumorigenic mechanisms such as cell cycle control, invasion, metastasis, and chemoresistance [11]. Evidence supporting the role of epigenetic alterations, including miRNA modulation, in PDAC pathogenesis is ever growing. Abnormal miRNA expression contributes to PDAC development through facilitating oncogene expression, such as miR-217 expression influencing *AKT*, or inhibiting tumor suppressors, as seen through the overexpression of miR-15a down-regulating *WNT3A* [12]. miRNAs can also be secreted into extracellular fluids and circulate freely, or via vesicles such as exosomes (Figure 1) [9]. Circulating miRNAs are attractive prospects for biomarkers due to their stability and ease of collection through a simple blood sample [13].

Advances in molecular techniques have facilitated the advent of novel nucleic acid biomarkers, over traditional protein-based markers, which can detect PDAC earlier and better inform clinicians with regard to altering patient treatment strategies, which would significantly increase survival time and reduce unnecessary toxicity (summarized in Table 1). Such advances include Droplet Digital PCR (ddPCR) [14]. Similar to real-time polymerase chain reaction (qPCR), ddPCR employs enzymatic amplification of a nucleic acid template in a primer/probe format. The primary differences between conventional qPCR and ddPCR are the separation of the individual PCR reactions into thousands of oil-enclosed droplets before amplification occurs, and the assignment of a positive/negative end-point threshold for each reaction, thereby allowing for the quantification of nucleic acids independent of PCR efficiency. The differences allow for direct and independent quantification of nucleic acids with greater precision and reproducibility in samples with extremely low target molecules, when compared with traditional qPCR [15]. This ability to detect genetic material at low concentrations enables the detection of mutations in tumor-released nucleic acids in circulation, despite the fact that these nucleic acids represent a small proportion of the total circulating genetic material (such as genomic and mitochondrial DNA). An alternative technique used for the detection of nucleic acid biomarkers is next-generation sequencing (NGS). NGS are new technologies that allow for DNA/RNA sequencing and mutation detection [16]. A variety of NGS platforms exist, utilizing different sequencing technologies; however, all platforms sequence millions of fragments of nucleic acid, with the resultant data produced analyzed using sophisticated bioinformatic pipelines. NGS begins with nucleic acid fragmentation, via mechanical or enzymatic methods, and subsequent isolation through labelling with specific complementary probes. PCR-based amplification can be performed on targeted DNA segments, with the resultant products used for library preparation. Library preparation involves the addition of adaptor molecules to the DNA fragments, which facilitates their attachment to the flow cell/chip on sequencing and also indexing for sample identification in multiplex assays. Sequencing principles vary between methods, such as the Illumina NGS which measures the emission of fluorescent tagged nucleotides as they are added to a template strand (sequencing by synthesis), or the Ion Torrent NGS which individually loads nucleotides to the strand and measures the release of a hydrogen ion when a nucleotide is incorporated (semiconductor sequencing) [17,18]. Regardless of the technique employed, the resultant data are compared to a reference genome for mutation/variant identification and sequence segments are amalgamated to generate sequencing results for the full length target DNA [16]. NGS allows for highly sensitive and accurate processing of circulating nucleic acids for high throughput detection of both hotspot and unknown mutations [19].

With such rapid advancements in the field of molecular diagnostics and the use of liquid biopsies, combined with a critical need for an improvement in the diagnostic capabilities for PDAC, a comprehensive review comparing and contrasting the various potential circulating nucleic acid biomarkers, with critical analysis of their clinical applications, is highly warranted. Novel biomarkers identified must provide relevant clinical use, with a significant gap currently existing between biomarker discovery and clinical utility [33].

## 2. Protein Biomarkers in PDAC

Carbohydrate Antigen 19-9 (CA 19-9) is synthesized at low levels in the pancreas and biliary tract, with normal circulating CA 19-9 levels of 0–37 U/mL [34,35]. An increase in expression outside this range is often associated with PDAC, with increasing CA 19-9 levels correlating with advanced stages [36]. CA 19-9 is currently the only biomarker approved by the National Comprehensive Cancer Network (NCCN) for use in the diagnosis and monitoring of PDAC [37]. Despite this, it has several significant limitations such as moderate sensitivity and specificity at an estimated 79% and 82%, respectively [38]. Additionally, approximately 5%–10% of the population is Lewis blood group negative and so secrete little to no CA 19-9 rendering this biomarker obsolete in these patients [39]. CA 19-9 is also elevated in various other conditions such as chronic pancreatitis and obstructive jaundice, which are included in the differential diagnosis of PDAC and so may cause difficulty during investigations [38]. CA 19-9 is also an unsuitable biomarker for screening purposes due to its poor positive predictive value (PPV) of approximately 0.9%, indicating it does not perform well in identifying PDAC in an asymptomatic population [40]. Furthermore, CA 19-9 has been shown to be ineffective in identifying small tumors (<3 cm) and cannot distinguish between PDAC and precursor lesions such as intraductal papillary mucinous neoplasms (IPMNs), which are also associated with elevated serum CA 19-9 levels [38,41,42].

Carcinoembryonic antigen (CEA) is the second most commonly used serum biomarker for the clinical diagnosis of PDAC; however, it is not recommended by the NCCN guidelines [43]. CEA is a fetal glycoprotein generally produced at insignificant quantities after birth; however, its secretion is associated with a variety of pathological conditions, including PDAC [44]. A meta-analysis of CEA’s performance as a biomarker in detecting PDAC found a relatively poor estimated sensitivity of 54%, and a specificity of 79% [45]. Elevated preoperative CEA (>4.45 ng/mL) in patients with known PDAC was found to be associated with earlier disease recurrence [46].

Cancer antigen 125 (CA-125) is an antigenic tumor marker most commonly associated with epithelial ovarian neoplasms, however it has also been found to be elevated in PDAC, with expression increasing throughout disease progression [47,48]. Lou et al., found CA-125 to be a superior biomarker in predicting patients’ eligibility for surgical resection than CA 19-9 (Sensitivity 79%, specificity 71%) [49]. An elevated CA-125 (<18.6 U/mL) was found to be a significant risk predictor of poor overall survival (OS) and disease-free survival (DFS) in preoperative PDAC patients [50].

## 3. *KRAS* Mutations in Cell Free DNA in Combination with Protein Biomarkers as a Diagnostic Panel

Cohen et al. investigated the use of a protein biomarker panel in combination with circulating tumor DNA (ctDNA) tests for the *KRAS* mutation in order to detect early stage PDAC [24]. PDAC is characterized by an accumulation of various genetic mutations, however the driving force in progression from early precancerous lesions (PanIN) to advanced disease are mutations in the *KRAS* oncogene. Oncogenic *KRAS* is identified in nearly 100% of PDAC patients where it confers constitutive activation of the *KRAS* protein, with downstream effects in cellular proliferation, migration and chemotherapy resistance [51]. Most mutations (70%–95%) occur in codon 12 of exon 2, resulting most commonly in the conversion of wild-type glycine (GGT) to aspartic acid (GAT) (40%), valine (GTT) (33%), or arginine (CGT) (15%). Point mutations can also occur, albeit less frequently, in codon 61 of exon 3, codon 13 of exon 2, and codon 117 and 146 of exon 4 [52]. These point mutations result in the inactivation of GTPase enzymes and thus constitutive Ras signaling, which promotes the activity of various downstream cascades; most commonly through RAF/MAPK, ERK1/2, PI3K, and Akt [6,53]. Mutant *KRAS* playing a major role in disease progression highlights it as an auspicious biomarker for PDAC. CA 19-9, while inadequate as a lone marker for diagnosing PDAC, may find utility in a panel for the early diagnosis of PDAC. A relatively high threshold for CA 19-9 PDAC positivity (100 U/mL) was chosen in order to facilitate its use for screening in a healthy population, where levels this high are seldom found. Twenty-nine protein biomarkers were subsequently evaluated for potential use in screening for pancreatic cancer, of those 29 biomarkers, five were found to be elevated in PDAC: carcinoembryonic antigen (CEA), hepatocyte growth factor (HGF), osteopontin (OPN), midkine, and prolactin. Midkine and prolactin were excluded from further analysis as they were found to be falsely elevated by anesthesia. Positive thresholds for the remaining three proteins (CEA, HGF, and OPN) were determined by 10% higher than the maximum value detected in the healthy control cohort. ctDNA can be single or double-stranded DNA which is released by tumor cells into circulation (Figure 1). Often tissue biopsies fail to capture the heterogeneity of potential mutations/biomarkers as they are not present uniformly in a tumor. As ctDNA is released directly by the tumor cells, they harbor identical mutations to the releasing cells, and thus can better represent the diversity of tumors [54]. A PCR-based “Safe-Sequencing System” (Safe-SeqS) was used to assess ctDNA for *KRAS* mutations at codon 12 exon 2 (p.G12A,c.35G>C; p.G12C,c34G>A; p.G12D,c.35G>A; p.G12V,c.35G>T), and codon 61 exon 3 (p.Q61H,c.138A>C; p.Q61H,c.138A>T), the two most common disease-associated variants. *KRAS* mutations were observed in 30% of PDAC cases, with greater frequency in stage II cases with larger tumors (Table 1) [24]. These observations support the findings of Mohan et al. where somatic *KRAS* mutations were observed in 25% of locally advanced disease using ddPCR; however, the detection rate was substantially increased through NGS analysis of an additional 640 cancer associated genes to 50% in locally advanced disease [28]. On average 5.3 mutant templates per milliliter of plasma was detected, highlighting the need for extremely sensitive techniques for detecting *KRAS* in ctDNA [24]. Perfect concordance between the mutation identified in patient’s plasma and that found in the primary tumor was observed. The protein biomarkers individually all displayed reduced sensitivity compared to *KRAS* ctDNA analysis alone, but demonstrated 100% specificity in the independent test cohort. A combination of *KRAS* ctDNA, CA 19-9, CEA, HGF, and OPN demonstrated a specificity of 99.5% and a sensitivity of 64%. Of patients in this study with PDAC that demonstrated no typical symptoms, this combination assay of ctDNA and protein biomarkers identified 60% of positive cases. Only early stage PDAC patients were included in this study as these patients would benefit the most from a screening program that could identify PDAC, i.e., in resectable disease, however this undoubtedly decreased the apparent sensitivity of this combination assay as both ctDNA and protein biomarkers have been shown to be increasingly elevated in late stage disease [24]. A screening panel of biomarkers such as this could be used in high-risk groups which are associated with increased incidence of PDAC such as new-onset diabetes or obesity. Allenson et al. demonstrated that in early stage disease, exosomal DNA analysis for *KRAS* mutations outperformed ctDNA, and so a future study substituting exosomal DNA analysis for ctDNA in a similar PDAC biomarker panel may improve its sensitivity [23]. An important caveat to this screening panel was that it was only employed in discriminating PDAC from healthy controls, its efficacy in differentiating PDAC from other pancreaticobiliary diseases such as chronic pancreatitis should also be evaluated, as these conditions also often result in increased levels of CA 19-9 [38]. Incidentally, many of these conditions are symptomatic and so this may also aid in differentiating them from PDAC.

## 4. miRNA as a Diagnostic Biomarker in Early-Stage Disease

Vila-Navarro et al. investigated a potential role for miRNAs in detecting PDAC [30]. Genome-wide profiling identified 607 significantly dysregulated miRNAs in PDAC samples, in addition to 396 miRNAs that were deregulated in IPMNs, which are potentially precancerous lesions. These data highlight the vast diversity between miRNA expression profiles in PDAC and healthy tissue. Interestingly 325 of the 396 miRNAs deregulated in IPMNs are common between the precursor lesions and PDAC. This indicates that abnormal miRNA expression begins early in the development of PDAC and could therefore be a significant driving factor. Of note is the fact that the majority of PDAC cases used in the generation of this PDAC miRNA profile were in the early stages (I and II) of development, further highlighting the early role of miRNA deregulation in PDAC. In endoscopic ultrasound-guided fine needle aspiration (EUS-FNA) samples, a cohort of 13 of these miRNAs (miR-93, miR-16, miR-548d-3p, miR-320a, miR4468, miR-3120–3p, miR-4713–5p, miR-103a, miR-155, miR4770, miR-181a, miR-221, and miR-151b) demonstrated the ability to discriminate PDAC from healthy controls with an area under the curve (AUC) of over 0.9 (Table 1) [30]. In fact, miR-93 alone demonstrated an AUC of 0.995 (95% CI 1.00–0.99), highlighting its significant potential as a biomarker for the differentiation of PDAC from normal pancreatic tissue. An important note however is that plasma miR-93 is also upregulated in type 2 diabetic retinopathy patients, as identified by Zou et al. [55]. As type 2 diabetes is a significant risk factor for PDAC, this could potentially limit the use of miR-93 alone in this particular cohort of patients and may be more appropriate for inclusion in a diagnostic panel. A panel of 5 miRNAs (miR-103a, miR-155, miR-181a, miR-181b, and miR-93) were able to identify IPMNs from controls with an AUC over 0.9 (Table 1) [30]. miR-103a, miR181a and miR-93 were also found to be significantly upregulated (3.43-, 2.57-, 3.79-fold change, respectively) in the serum of patients with IPMNs compared to controls in a separate study [56]. IPMNs demonstrate considerable ability to progress into PDAC, therefore the identification of a biomarker to differentiate them from normal pancreatic tissue would be a valuable addition to current diagnostic panels, allowing for early monitoring of high-risk patients.

An investigation into the use of circulating miRNA as a potential diagnostic tool for detecting early stage PDAC in plasma samples yielded promising results [32]. miRNA profiling of 136 stage II PDAC cases and 73 controls, identified three significantly deregulated miRNAs (miR-34a-5p, miR-130a-3p, and miR-222-3p) in PDAC cases compared to controls. While the three identified miRNAs did not outperform CA 19-9 using Receiver Operating Characteristic (ROC) analysis, a combination of each miRNA with CA 19-9 improved upon CA 19-9s AUC from 0.89 (95% CI, 0.81–0.95) alone to 0.92 (95% CI, 0.86–0.97), 0.94 (95% CI, 0.89–0.98), and 0.92 (95% CI, 0.87–0.97), respectively (Table 1). miR-34a’s link to PDAC has been previously identified, with downregulation of miR-34a being associated with an increase in metastatic characteristics such as invasion, angiogenesis, and migration through signaling pathways such as Notch1 and JAK2/STAT3 [57,58]. Similarly, both miR-34a-5p and miR-130a-3p were identified as promotors of PDAC progression via PI3K/AKT signaling resulting in mesenchymal-to-epithelial transition (MET); the post-EMT retransition to an epithelial phenotype to allow anchorage of metastatic cells for colonization [59,60]. miR-222 has also been implicated in PDAC metastasis through AkT activation and p27 phosphorylation, which influences key processes such as cytoskeletal remodeling and cellular plasticity [61,62]. The identification of these miRNAs as potential biomarkers in early-stage PDAC is thus surprising given metastasis is more commonly associated with advanced disease. Determining if these biomarkers can predict metastasis may identify patients with early-stage disease at risk of developing metastatic disease and thus requiring an otherwise alternative or adjuvant therapeutic intervention, allowing for more targeted therapy in early-stage disease with increased probability of metastasis.

## 5. Potential of Exosomal miRNA as a PDAC Biomarker

An alternative approach in analyzing miRNA deregulation in PDAC is the evaluation of exosomal miRNA signatures in patient serum to identify PDAC. Exosomes are a membrane bound subtype of extracellular vesicle (alongside microvesicles and apoptotic bodies) that are secreted by a variety of cell types such as endothelial, epithelial, immune, and cancer cells (Figure 1) [63,64]. Importantly, studies have demonstrated that neoplastic cells produce greater volumes of exosomes than healthy tissues, indicating that isolation of these exosomes from circulation and subsequent analysis of exosomal content, such as miRNAs and genomic DNA, may prove to be valuable biomarkers [65]. In an in vitro study, Xu et al. identified significantly increased expression of exosomal miR-196a and miR-1246 secreted from pancreatic adenocarcinoma cells (PANC-1) compared to normal pancreatic epithelium (hTERT-HPNE) [21]. Furthermore, a cohort of patients with localized pancreatic cancer (stage I/IIa) demonstrated significantly elevated plasma exosomal miR-196a and miR-1246 compared to matched healthy controls. Receiver operating characteristic curves for miR-196a and miR-1246 in the diagnosis of localized pancreatic cancer demonstrated reasonable AUCs of 0.81 (95% CI 0.64, 0.97; *p* < 0.001) and 0.73 (95% CI 0.54, 0.92; *p* = 0.019) respectively (Table 1). Further analysis into the discriminatory power of the exosomal miRNAs indicate that miR-1246 is an exceptional marker for distinguishing IPMNs from healthy controls (*p* < 0.0001) and miR-196a is more suited for differentiating localized PDAC from controls (*p* = 0.0053). While this study identifies two promising miRNAs for future use as biomarkers in PDAC, their sensitivity and specificity must be improved for adoption into clinical use. Incorporating novel miRNA biomarkers such as these with alternative markers such as *KRAS* cell free/exosomal DNA and existing protein biomarkers such as CA 19-9 may allow for the formation of a diagnostic panel with improved discriminatory power.

An alternative approach was taken by Nakamura et al. where they analyzed pancreatic juice, rather than serum, for exosomal miRNA biomarkers for the detection of PDAC [20]. In cases where EUS-FNA is not advised due to risk of tumor cell transmission, cytological evaluation of tumor cells in the pancreatic juice can be performed via endoscopic retrograde pancreatography (ERP). In such cases, the pancreatic juice supernatant is discarded and only the cellular content is analyzed, however the pancreatic juice contains exosomes secreted by the tumor, which may contain valuable markers such as miRNAs. Exosomal miRNA levels in pancreatic juice for ex-miR-21 and ex-miR-155 were significantly higher in PDAC patients compared to chronic pancreatitis (*p* = 0.0006 and *p* = 0.008, respectively). Due to the invasive nature of EUS-FNA, no healthy controls were included in this study, further highlighting the benefit of a serum based liquid biopsies for screening purposes (Table 1). No such correlation was observed for free miR-21 or miR-155 (*p* = 0.08 and *p* = 0.61, respectively). In fact, when stored at 37 °C, physiological temperature for pancreatic juice, free miRNA was shown to decrease over time while exosomal miRNA did not, most likely due to the enclosed nature of the exosome conferring protection form RNases. The diagnostic capabilities of ex-miR-21 and ex-miR-155 were superior to that of CA 19-9 (AUC = 0.90 and 0.89 versus 0.68). In addition, ex-miR-21 and ex-miR-155 displayed greater accuracy in detecting PDAC compared to pancreatic juice cytology (ex-miR-21 = 83%, ex-miR-155 = 89%, PJC = 74%). A combined test of either ex-miR-21/ex-miR-155 and pancreatic juice cytology results in an impressive sensitivity of 93% and specificity of 88%. The primary limitation of analyzing exosomal miRNA in pancreatic juice as a biomarker for PDAC is the relatively invasive nature of the ERP procedure, which is far less convenient than the collection of a blood sample for analyzing circulating free miRNAs, and is associated with iatrogenic acute pancreatitis [66].

## 6. Exosomal Derived *KRAS* Mutations as a Diagnostic Biomarker

Similar to miRNA, exosomes also contain DNA exocytosed from the tumor cells. ddPCR analysis of exosomal DNA identified *KRAS* codon 12/13 mutations (lower limit for mutant allele frequencies at 0.01% considered positive) in 66.7%, 80%, and 85% of localized, locally advanced and metastatic PDAC patients’ serum respectively [23]. Exosomal *KRAS* status could predict disease progression with sensitivity and specificity of 75.4% and 92.6% respectively, with a positive exosomal *KRAS* result indicating an 8.17 times greater probability of having localized pancreatic cancer (Table 1). An important note however is the fact that a positive exosomal *KRAS* mutation was detected in 7.4% of healthy controls. For appropriate use of *KRAS* mutation status to be used as a diagnostic tool in PDAC, ‘background’ mutation rates must be better classified to allow for a lower limit of *KRAS* mutation allele frequency facilitating better discrimination of normal and oncogenic mutation rates. Interestingly, *KRAS* mutation status in healthy controls was positively associated with patient age, indicating a potential for detecting *KRAS* mutations in the premalignant stages of PDAC or an alternative malignancy also associated with *KRAS* mutagenesis, such as other adenocarcinomas of the lung or colon [23,67,68]. Localized PDAC patients stratified by an exosomal *KRAS* mutation allele frequency of <1% pre-surgery was associated with longer disease-free survival of patients at a median disease-free time of 441 days compared to only 127 days in patients sorted by a mutation allele frequency of >1%. These results indicate that exosomal *KRAS* status may be a potential biomarker to indicate the need for more aggressive adjuvant therapeutics, such as modified-FOLFIRINOX [69]. Interestingly, while only a minor statistically significant positive correlation was observed between *KRAS* mutation allele frequency and CA 19-9, no correlation was found between disease-free survival and CA 19-9, further highlighting its inadequacies as a biomarker in PDAC. Allenson et al. also investigated ctDNA in PDAC patient serum for *KRAS* mutations. Interestingly, strong concordance between *KRAS* detection in exosomal DNA and ctDNA was observed in late stage PDAC but in earlier stages of disease, exosomal DNA detected *KRAS* mutations at a higher rate. A possible explanation for this discrepancy is the fact that while exosomal DNA is released from tumor cells by endocytosis, ctDNA is released during apoptosis, which occurs at a higher rate in more advanced disease [23].

## 7. Circulating Nucleic Acids as Prognostic Biomarkers

A primary cause of the poor clinical outcomes associated with PDAC is the poor efficacy of treatment due to multi-drug resistance, however the use of predictive biomarkers to identify patient response to therapy and prognosis is not standardized. The use of circulating nucleic acids have been identified as promising potential prognostic biomarkers to enhance therapeutic performance. Bernard et al. investigated how *KRAS* mutation status in both ctDNA and exosomal DNA through ddPCR could be used as prognostic markers in PDAC through longitudinal monitoring [22]. *KRAS* mutations were identified in exosomal DNA at higher rates than ctDNA in both metastatic (61% versus 53%) and locally advanced diseases (38% versus 34%). Higher concordance of *KRAS* mutation status between liquid biopsy and surgically resected tumors was observed in exosomal DNA (95.5%) compared to ctDNA (68.2%). Exosomal *KRAS* mutant allele fraction was shown to be a predictive marker of candidates for surgical resection with an increase of exosomal *KRAS* MAF (Mutant Allele Frequency) at completion of neo-adjuvant therapy indicating a patient is not a suitable candidate for resection, and the inverse being true for patients with a reduction in exosomal *KRAS* MAF presenting as candidates for surgical resection (odds ratio 38.4, *p* = 0.0002) (Table 1). While no such correlation was seen for ctDNA, CA 19-9 was also significantly associated with surgical resection candidates (odds ratio 28.0, *p* = 0.003), indicating a potential complementary use of both biomarkers; where exosomal DNA would be more appropriate in CA 19-9 nonexpressers or where comorbidities result in unreliable CA 19-9 quantification, and CA 19-9 could be used where exosomal DNA *KRAS* mutations are undetectable [22].

In a longitudinal study performed by Bernard et al., ctDNA and exosomal DNA *KRAS* mutations were significantly associated with a reduction in both progression free survival (PFS) and OS, with any detectable ctDNA associated with a reduced PFS and OS, and exosomal *KRAS* MAF > 5% being associated with shorter PFS and OS [22]. The association between *KRAS* ctDNA and PFS/OS was also established by Del Re et al. where an increase or decrease in mutant *KRAS* ctDNA at day 15 of treatment compared to baseline was correlated with an inverse change in PFS or OS [27]. Similarly, *KRAS* copy number gain was identified as a significant factor associated with shorter survival times (*p* < 0.05) in both patients with locally advanced and metastatic disease [28]. Additionally, both detectable ctDNA and exosomal DNA *KRAS* MAF > 5% in the same patient were indicators of even poorer OS, highlighting the complementary nature of these markers [22]. The utility of these markers as predictors of outcomes may facilitate the selection of candidates for more aggressive therapy or closer monitoring to enable earlier detection of regression. A significant association between exosomal DNA *KRAS* MAF and disease progression of patients undergoing treatment was established, with a MAF peak > 1% demonstrating considerable ability to predict progression with sensitivity of 79% and specificity of 100% [22]. While CA 19-9 could also predict progression, albeit with lower sensitivity and specificity (70% and 89% respectively), only exosomal *KRAS* could predict disease progression before it was clinically identifiable through computerized tomography (CT) scan, with a median lead time of 50 days. This spike in exosomal DNA *KRAS* MAF is likely due to the development of treatment resistance and a resultant increase in tumor growth, therefore this early indication would allow for a change of therapeutic regime, preventing unnecessary toxicity and enabling more efficacious treatment. No significant correlation was observed between ctDNA MAF and disease progression [22]. This contrasts with the findings of Del Re et al., where all patients with an increase in *KRAS* ctDNA from baseline during treatment had disease progression at 2-month radiological evaluation [27]. A potential explanation for this discrepancy is the fact that while Bernard et al. used ctDNA MAF as a marker (i.e., the ratio of mutant to wild type allele), Del Re et al. stratified patients by an increase in mutant *KRAS* ctDNA [22,27].

Nakano et al. found the detection of ctDNA *KRAS* mutations in preoperative and postoperative PDAC patient serum to be predictive markers of survival and response to treatment [26]. Patients that transform from ctDNA *KRAS* negative preoperatively to ctDNA *KRAS* positive postoperatively had significantly shorter DFS and OS than those that remained negative postoperatively (Table 1). Additionally, a significant positive correlation was identified between early disease recurrence and a shift from mutation-negative to mutation-positive postoperatively. These results indicate that ctDNA is a potential biomarker to monitor the response of patients to curative resection and to predict disease outcome. The shift from negative to positive *KRAS* mutation status postoperatively is unexpected. Potential explanations include ctDNA release from malignant cells during surgery due to tumor manipulation or the release of ctDNA from metastatic lesions that were undetected by preoperative imaging, which would explain why the shift to mutation-positive was correlated with worse outcomes [70]. A comparison between the *KRAS* mutation status of the tumor material excised during surgery and the postoperative ctDNA in this patient cohort may aid in further elucidating the underlying mechanism governing the shift from mutation negative to positive.

ctDNA has a relatively short half-life of approximately 2 h compared to protein biomarkers such as CA 19-9, which has a half-life of 0.5 days in the central compartment (plasma) and 4.3 days in the peripheral compartment (tissues) [71,72]. This shorter half-life of ctDNA lends itself to use as a short-term dynamic biomarker. Such a utility was investigated by Perets et al. where the slope of *KRAS* ctDNA levels in PDAC patients was calculated as the change in *KRAS* ctDNA level of consecutive samples over the difference between sample times [25]. A significant correlation was found between ctDNA slopes and survival times (r = −0.76, *p* = 0.03), where a quick and sharp decline in ctDNA expression was associated with improved survival times, and a rapid and marked increase in ctDNA expression correlated with a worse prognosis (Table 1). Unsurprisingly, CA 19-9 slope demonstrated no significant correlation with survival time. The use of dynamic *KRAS* ctDNA monitoring in PDAC could be of particular value in monitoring the immediate effects of an intervention such as surgery or initiation of a new therapeutic, allowing for rapid alterations in treatment where the desired response is not obtained.

## 8. miRNA in Detecting Lymph Node Metastasis

In PDAC, lymph node metastasis is significantly associated with a worse prognosis, particularly following curative surgery [73]. Preoperative staging of lymph node metastasis relies on imaging studies, which have suboptimal precision [29]. Postoperative lymph node staging is dependent on extent of lymphadenectomy and pathological examination, both of which are operator dependent and, as such, subject to variable dependency. Consistent and accurate biomarkers for the detection of metastasis would greatly improve treatment utility in PDAC patients. Patients with distant metastasis could avoid unnecessary intervention while patients with nodal spread may avail of a more aggressive therapeutic regime.

Lemberger et al. investigated a potential association between nodal metastasis and miRNA expression in PDAC patients [29]. A panel of 6 miRNAs (miR-141, miR-155, miR-720, miR-216a, miR-196a and miR-130b) associated with different patterns of expression between PDAC patients with and without lymph node metastasis was formed using microarray analysis of formalin fixed paraffin embedded PDAC tissue (Table 1). Using a scoring system based on aberrant expression of each of the 6 markers in the panel, a score of >4 was highly predictive of nodal metastasis with positive and negative predictive values of 83.7% and 70.5% respectively. Circulating miRNA expression was also assessed in the plasma of PDAC patients with and without nodal metastasis using qPCR. PDAC patients with nodal metastasis demonstrated higher expression of miR-196a and miR-155 (2.585 and 1.596 fold respectively), and significantly lower expression of miR-720 and miR-141 (2.268 and 1.651 fold respectively) compared to plasma of PDAC patients without nodal metastasis [29]. miR-155 has previously been linked to PDAC through its increased detection in pancreatic juice exosomes compared to chronic pancreatitis. It has also been reported that miR-155 plays a role in gemcitabine resistance through exosome formation, by decreasing levels of inhibitors of the RAB family of genes, which are key exosome regulators [74]. miR-155 has also been implicated in anti-apoptotic activity in gemcitabine resistant PDAC through down regulation of its tumor suppressor target genes, which include *Sel-1-like*, and *TP53INP1* [74]. Similarly, miR-196a has been demonstrated to be elevated in plasma exosomes of PDAC patients, with a significant elevation of plasma ex-miR-196a in more advanced tumors (stages III and IV) compared to locally advanced PDAC (stages I and II) [75]. Furthermore, ex-miR-196a levels demonstrated ability to predict overall survival when PDAC patients were divided into high- and low-level groups (6.1 months 95% CI, 4.49–7.72 versus 12 months 95% CI, 5.92–18.08, *p* = 0.007). In vitro analysis of the PANC-1 pancreatic ductal adenocarcinoma cell line transfected with chemically synthesized miR-141 and miR-720 indicated that they play a role in increasing expression of E-cadherin, a cellular adhesion protein, through decreased levels of key regulators such as ZEB-1 and TWIST-1 [29]. This correlates with expression levels demonstrated in tissue and blood samples from PDAC patients with nodal metastasis where decreased expression of miR-141 and miR-720 was associated with epithelial to mesenchymal transition, which allows for the detachment of cancer cells from the primary tumor and thus facilitates metastasis [76].

A retrospective study into serum miRNA profiles of PDAC patients conducted by Aita et al. identified eight miRNAs (miR-1915-3p, miR-371b-5p, miR-1202, miR-4669, miR-3679-5p, miR-6088, miR-4499, and miR-7107-5p) to be significantly associated with lymph node metastasis (Table 1) [31]. Of particular note from this group are miR-4669 and miR-1202, both of which displayed decreased expression in cases where lymph node metastasis occurred. miR-4669 expression has also been identified in colon cancer, where enhanced expression was observed in cases with no lymph node involvement, and reduced expression in lymphatic metastasis cases [77]. Altered miR-1202 expression has been associated with a variety of cancers. Similar to the findings of Aita et al., miR-1202 expression was decreased in cervical cancer, with further reductions in expression associated with later clinical staging [78]. miR-1202′s potential role as a tumor suppressor was investigated in glioma cells where restoration of its expression resulted in an inhibition of proliferation and increased apoptosis through small GTPase Rab1A [79]. Rab1A has also been associated with cellular adhesion and migration [80]. These results indicate miR-1202 may play a role as a tumor suppressor in PDAC, although the specific signaling mechanisms through which it functions must first be further elucidated.

## 9. Combined Diagnostic Panel

Yang et al. developed a blood-based biomarker panel using machine learning to improve diagnosis and staging of PDAC [81]. Feature selection using Least Absolute Shrinkage and Selection Operator (LASSO) was performed on 14 candidate biomarkers (6 exosomal mRNAs, 5 exosomal miRNAs, ctDNA *KRAS* MAF, circulating cell free DNA concentration and CA 19-9) to identify, train and validate optimal biomarker panels for distinguishing PDAC from non-cancer controls and for the differentiation of metastatic from non-metastatic PDAC. A panel of 5 biomarkers with the best performance (AUC = 0.93, sensitivity 88% and specificity 95%) for diagnosing PDAC were selected which consisted of exosomal mRNA CK18 and CD63, exosomal miR-409, circulating ctDNA concentration and CA 19-9. CK18 is a structural protein which is associated with carcinogenesis through several signaling cascades such as PI3K/Akt, Wnt, and ERK/MAPK signaling, which are involved in regulation of cellular proliferation, apoptosis and motility [82]. CD63 is an established exosomal marker with a significant correlation with PFS and OS in PDAC [83]. Reduced expression of miR-409 has been observed in a variety of cancers such colorectal, non-small-cell lung, and osteosarcoma, where it inhibits proliferation, invasion and tumorigenesis, implicating a role as a tumor suppressor [84,85,86]. An alternate panel was constructed for the differentiation of metastatic from non-metastatic PDAC. The goal of this panel was to be used in conjunction with standard imaging procedures to aid in further identifying patients classified by imaging as being metastasis free who in fact have occult metastasis. This panel outperformed imaging alone with an accuracy of 84%, compared to 64% for imaging. The biomarker panel used consisted of 4 markers: exosomal miR-1299, exosomal mRNA *GAPDH*, ctDNA *KRAS* MAF, and CA 19-9. miR-1299 has been identified as a tumor suppressor in prostate cancer through the inhibition of cellular proliferation and metastasis [87]. Similarly, it has recently been shown to be downregulated in PDAC and so potentially functions as a tumor suppressor in a similar fashion [88]. Mutations in the tumor suppressor gene *TP53*, which is implicated in approximately 70% of PDACs, results in dysregulation of GAPDH nuclear translocation. Maintenance of GAPDH in the cytosol plays a central role in the mutant *P53* mediated inhibition of apoptosis and development of autophagy-induced gemcitabine resistance [89]. These findings demonstrate the complementary nature of current imaging techniques and established biomarkers alongside novel biomarkers to further improve the diagnostic and staging capabilities of blood-based assays.

## 10. Conclusions

Considerable advances in our ability to detect nucleic acids in liquid biopsies through novel techniques have allowed for the detection of select RNA profiles associated with PDAC and for circulating DNA with the essentially ubiquitous *KRAS* mutation. Current diagnosis and monitoring of PDAC is reliant on inadequate biomarkers and subjective imaging which fails to detect developments in tumor growth and response to treatment in ample time to allow for informed clinical decisions. The analysis of circulating nucleic material in liquid biopsies may present an opportunity to significantly improve the currently dismal mortality rate associated with PDAC through their use in the diagnosis, prediction of treatment response, and prognosis of PDAC. This review provides a comprehensive overview of the promise and potential of liquid biopsies for cancer and highlights their utility for the diagnosis, prognosis, disease stratification and therapeutic monitoring of PDAC. Circulating nucleic acids have been demonstrated to present a valuable addition to the currently limited repertoire of clinically available biomarkers. Further investigation into combination panels consisting of molecular markers, novel protein markers and established assays such as CA 19-9 quantification is required to allow for their incorporation into clinical application, but promising studies have outlined potential candidate-biomarkers for inclusion in such panels, along with some potential panels requiring further validation. Clinical trials validating these promising biomarkers through standardized methods is required in order to substantiate the utility of such markers in PDAC. Highly sensitive molecular techniques are required in order to detect mutations in circulating nucleic acids, and while such assays do exist (such as ddPCR and NGS), they are yet to be established within most routine clinical laboratories. For full integration of such techniques into clinical settings a reduction in the costs and timescale associated with generation and interpretation of the vast amount of data associated with such assays is necessary. This novel area of molecular diagnostics shows considerable promise, but is not without significant challenge.

## Figures and Tables

**Table 1:** Summary of Nucleic Acid Biomarkers for PDAC.

Biomarker	Patient Cohort	Method	Significant Comments	Ref.
**Exosomal miR-21, -155**	PDAC (*n* = 27), Chronic Pancreatitis (*n* = 8)	qPCR	Exosomal miR-21 and Exosomal miR-155 in pancreatic juice samples could differentiate PDAC from CP with significant AUC.	[20]
**Exosomal miR-196a, -1246**	Localized PDAC (*n* = 15), HC (*n* = 15)	qPCR	Exosomal miR-196a and miR-1246 could discriminate between PDAC and controls with AUCs of 0.81 and 0.73 respectively. miR-196a was a better indicator of PDAC, whereas miR-1246 was a better indicator of IPMNs (*p* = 0.0053, and *p* < 0.0001 respectively).	[21]
**Exosomal *KRAS* codon 12/13 mutations**	PDAC (*n* = 194), Disease Controls (*n* = 37)	ddPCR	Exo*KRAS* level significantly correlated with candidates for surgical resection (OR = 38.4), and is an independent prognostic marker for PFS and OS (HR = 2.28 and 3.46 respectively). Exo*KRAS* could predict disease progression at significantly earlier times than both CA 19-9 and radiological imaging (sensitivity & specificity 79% & 100% respectively).	[22]
**Exosomal *KRAS* codon 12/13 mutations**	PDAC (*n* = 127), HC (*n* = 136)	ddPCR	Exo*KRAS* detected at a higher rate than ctDNA *KRAS* across all PDAC stages. Exo*KRAS* could predict PDAC with a sensitivity and specificity of 75.4% and 92.6% respectively. Pre-surgery exo*KRAS* MAF of <1% was associated with disease-free survival post resection.	[23]
**ctDNA *KRAS* codon 12/61 mutations**	Resectable PDAC (*n* = 221), HC (*n* = 182)	PCR-based SafeSeqS	*KRAS* mutations were identified in 30% of PDAC cases, more frequently in stage II and larger tumors than smaller stage I tumors. Agreement between mutations identified in ctDNA and in the primary tumor was 100%.	[24]
**ctDNA *KRAS* codon 12 mutations**	Metastatic PDAC (*n* = 17)	Ion Torrent PGM sequencer	Significant and rapid increase in ctDNA levels was associated with poor prognosis, and a sudden significant decrease in ctDNA was associated with an improved prognosis (r = −0.76, *p* = 0.03).	[25]
**ctDNA *KRAS* codon 12/13 mutations**	PDAC (*n* = 45)	qPCR with PNA Clamping	Postoperative detection of ctDNA *KRAS* is a prognostic marker for DFS (HR = 2.919). Transformation from pre-op KRAS negative to post-op KRAS positive indicated poor OS (HR = 9.419).	[26]
**ctDNA *KRAS* codon 12/13 mutations**	PDAC (*n* = 27)	ddPCR	Increase in ctDNA *KRAS* level during treatment was associated with decreased PFS and OS (median PFS 2.5 vs. 7.5 months, median OS 6.5 vs. 11.5 months).	[27]
**ctDNA *KRAS* codon 12/13 mutations**	Metastatic PDAC (*n* = 31), Locally Advanced PDAC (*n* = 24)	ddPCR	Best prognosis was identified in patients with wild-type *KRAS*, followed by *KRAS* mutation with no copy number gain, worst prognosis was associated with increasing *KRAS* mutation copy number (median survival 10.6, 5.5, and 2.5 months respectively).	[28]
**miR-155, -196a, -720, -141**	Nodal Metastasis PDAC (*n* = 10), No Metastasis PDAC (*n* = 10), HC (*n* = 10)	qPCR	Significantly higher expression of miR-155, miR-196a and lower expression of miR-720, miR-141 in PDAC with nodal metastasis versus without. Upregulation of miR-720, miR-141 resulted in decreased cellular aggressiveness and increased chemosensitivity in PDAC cell lines.	[29]
**miR-93, -16, -548d-3p, -320a, -4468, -3120–3p, -4713–5p, -103a, -155, -4770, -181a, -221, -151b**	PDAC (*n* = 60), HC (*n* = 26)	qPCR	13 miRNAs in EUS FNA samples could distinguish PDAC from controls with high accuracy (AUC > 0.9).	[30]
**miR-103a, -155, -181a, -181b, -93**	IPMN (*n* = 9), HC (*n* = 26)	qPCR	5 miRNAs in EUS FNA samples could distinguish IPMNs from controls with high accuracy (AUC > 0.9).	[30]
**miR-1915-3p, -371b-5p, -1202, -4669, -3679-5p, -6088, -4499, -7107-5p**	Stage I-III PDAC (*n* = 15), HC (*n* = 4)	Agilent Microarray	8 miRNAs significantly associated with lymph node metastasis. Of note miR-4669 and miR-1202, displayed decreased expression in cases where lymph node metastasis occurred.	[31]
**miR-34a-5p, -130a-3p, -222-3p**	Stage II PDAC (*n* = 136), HC (*n* = 73)	Abcam Fireplex-Oncology Panel	Combination miRNA with CA 19-9 improved upon CA 19-9s diagnostic ability. All 3 miRNAs identified are associated with metastasis.	[32]

HC: Healthy Control.

## Abbreviations

**Abbreviation**	**Description**
AkT	Protein Kinase B
AUC	Area Under the Curve
CA 19-9	Carbohydrate Antigen 19-9
CEA	Carcinoembryonic Antigen
CI	Confidence Interval
CK18	Cytokeratin-18
CT	Computed Tomography
ctDNA	Circulating Tumor DNA
ctRNA	Circulating Tumor RNA
ddPCR	Droplet Digital PCR
DFS	Disease Free Survival
EMT	Epithelial–Mesenchymal Transition
ERK	Extracellular-Signal-Regulated Kinase
ERP	Endoscopic Retrograde Pancreatography
EUS-FNA	Endoscopic Ultrasound Fine Needle Aspiration
Ex-miRNA	Exosomal Micro RNA
GAPDH	Glyceraldehyde 3-Phosphate Dehydrogenase
GTP	Guanosine-5’-triphosphate
HGF	Hepatocyte Growth Factor
IPMNs	Intraductal Papillary Mucinous Neoplasm
JAK	Janus Kinase
*KRAS*	Kirsten Rat Sarcoma Viral Oncogene Homolog
LASSO	Least Absolute Shrinkage and Selection Operator
MAF	Mutant Allele Frequency
MAPK	Mitogen-Activated Protein Kinase
MET	Mesenchymal-Epithelial Transition
miRNA	Micro RNA
mRNA	Messenger RNA
NGS	Next-Generation Sequencing
OPN	Osteopontin
OS	Overall Survival
PanIN	Pancreatic Intraepithelial Neoplasia
PDAC	Pancreatic Ductal Adenocarcinoma
PFS	Progression Free Survival
PGM	Personal Genome Machine
PI3K	Phosphatidylinositol-3-Kinase
PJC	Pancreatic Juice Cytology
PNA	Peptide Nucleic Acid
PPV	Positive Predictive Value
qPCR	Real-Time Polymerase Chain Reaction
RAF	Rapidly Accelerated Fibrosarcoma
ROC	Receiver Operating Characteristic
STAT	Signal Transducer and Activator
TME	Tumor Microenvironment
UTR	Untranslated Region
ZEB-1	Zinc Finger E-Box Binding Homeobox 1

## References

[1] Rawla P., Sunkara T., Gaduputi V. (2019). Epidemiology of Pancreatic Cancer: Global Trends, Etiology and Risk Factors. World J. Oncol..

[2] Bekkali N.L.H., Oppong K.W. (2017). Pancreatic ductal adenocarcinoma epidemiology and risk assessment: Could we prevent? Possibility for an early diagnosis. Endosc. Ultrasound.

[3] Garrido-Laguna I., Hidalgo M. (2015). Pancreatic cancer: From state-of-the-art treatments to promising novel therapies. Nat. Rev. Clin. Oncol..

[4] Matsuno S., Egawa S., Fukuyama S., Motoi F., Sunamura M., Isaji S., Imaizumi T., Okada S., Kato H., Suda K. (2004). Pancreatic Cancer Registry in Japan: 20 years of experience. Pancreas.

[5] Wang Z., Li Y., Ahmad A., Banerjee S., Azmi A.S., Kong D., Sarkar F.H. (2011). Pancreatic cancer: Understanding and overcoming chemoresistance. Nat. Rev. Gastroenterol. Hepatol..

[6] Javadrashid D., Baghbanzadeh A., Derakhshani A., Leone P., Silvestris N., Racanelli V., Solimando A.G., Baradaran B. (2021). Pancreatic Cancer Signaling Pathways, Genetic Alterations, and Tumor Microenvironment: The Barriers Affecting the Method of Treatment. Biomedicines.

[7] Karamitopoulou E. (2019). Tumour microenvironment of pancreatic cancer: Immune landscape is dictated by molecular and histopathological features. Br. J. Cancer.

[8] Vidigal J.A., Ventura A. (2015). The biological functions of miRNAs: Lessons from in vivo studies. Trends Cell Biol..

[9] O’Brien J., Hayder H., Zayed Y., Peng C. (2018). Overview of MicroRNA Biogenesis, Mechanisms of Actions, and Circulation. Front. Endocrinol..

[10] Di Leva G., Croce C.M. (2013). miRNA profiling of cancer. Curr. Opin. Genet. Dev..

[11] Bartel D.P. (2004). MicroRNAs: Genomics, biogenesis, mechanism, and function. Cell.

[12] Tesfaye A.A., Azmi A.S., Philip P.A. (2019). miRNA and Gene Expression in Pancreatic Ductal Adenocarcinoma. Am. J. Pathol..

[13] Cortez M.A., Bueso-Ramos C., Ferdin J., Lopez-Berestein G., Sood A.K., Calin G.A. (2011). MicroRNAs in body fluids—The mix of hormones and biomarkers. Nat. Rev. Clin. Oncol..

[14] Hindson B.J., Ness K.D., Masquelier D.A., Belgrader P., Heredia N.J., Makarewicz A.J., Bright I.J., Lucero M.Y., Hiddessen A.L., Legler T.C. (2011). High-Throughput Droplet Digital PCR System for Absolute Quantitation of DNA Copy Number. Anal. Chem..

[15] Taylor S.C., Laperriere G., Germain H. (2017). Droplet Digital PCR versus qPCR for gene expression analysis with low abundant targets: From variable nonsense to publication quality data. Sci. Rep..

[16] Qin D. (2019). Next-generation sequencing and its clinical application. Cancer Biol. Med..

[17] Gonzaga-Jauregui C., Zepeda Mendoza C.J., Gonzaga-Jauregui C., Lupski J.R. (2021). Chapter 4-Genomic sequencing of rare diseases. Genomics of Rare Diseases.

[18] Meera Krishna B., Khan M.A., Khan S.T., Tripathi V., Kumar P., Tripathi P., Kishore A., Kamle M. (2019). Next-Generation Sequencing (NGS) Platforms: An Exciting Era of Genome Sequence Analysis. Microbial Genomics in Sustainable Agroecosystems: Volume 2.

[19] Reuter J.A., Spacek D.V., Snyder M.P. (2015). High-throughput sequencing technologies. Mol. Cell.

[20] Nakamura S., Sadakari Y., Ohtsuka T., Okayama T., Nakashima Y., Gotoh Y., Saeki K., Mori Y., Nakata K., Miyasaka Y. (2019). Pancreatic Juice Exosomal MicroRNAs as Biomarkers for Detection of Pancreatic Ductal Adenocarcinoma. Ann. Surg. Oncol..

[21] Xu Y.-F., Hannafon B.N., Zhao Y.D., Postier R.G., Ding W.-Q. (2017). Plasma exosome miR-196a and miR-1246 are potential indicators of localized pancreatic cancer. Oncotarget.

[22] Bernard V., Kim D.U., San Lucas F.A., Castillo J., Allenson K., Mulu F.C., Stephens B.M., Huang J., Semaan A., Guerrero P.A. (2019). Circulating Nucleic Acids Are Associated With Outcomes of Patients With Pancreatic Cancer. Gastroenterology.

[23] Allenson K., Castillo J., San Lucas F.A., Scelo G., Kim D.U., Bernard V., Davis G., Kumar T., Katz M., Overman M.J. (2017). High prevalence of mutantKRAS in circulating exosome-derived DNA from early-stage pancreatic cancer patients. Ann. Oncol..

[24] Cohen J.D., Javed A.A., Thoburn C., Wong F., Tie J., Gibbs P., Schmidt C.M., Yip-Schneider M.T., Allen P.J., Schattner M. (2017). Combined circulating tumor DNA and protein biomarker-based liquid biopsy for the earlier detection of pancreatic cancers. Proc. Natl. Acad. Sci. USA.

[25] Perets R., Greenberg O., Shentzer T., Semenisty V., Epelbaum R., Bick T., Sarji S., Ben-Izhak O., Sabo E., Hershkovitz D. (2018). Mutant KRAS Circulating Tumor DNA Is an Accurate Tool for Pancreatic Cancer Monitoring. Oncologist.

[26] Nakano Y., Kitago M., Matsuda S., Nakamura Y., Fujita Y., Imai S., Shinoda M., Yagi H., Abe Y., Hibi T. (2018). KRAS mutations in cell-free DNA from preoperative and postoperative sera as a pancreatic cancer marker: A retrospective study. Br. J. Cancer.

[27] Del Re M., Vivaldi C., Rofi E., Vasile E., Miccoli M., Caparello C., d’Arienzo P.D., Fornaro L., Falcone A., Danesi R. (2017). Early changes in plasma DNA levels of mutant KRAS as a sensitive marker of response to chemotherapy in pancreatic cancer. Sci. Rep..

[28] Mohan S., Ayub M., Rothwell D.G., Gulati S., Kilerci B., Hollebecque A., Sun Leong H., Smith N.K., Sahoo S., Descamps T. (2019). Analysis of circulating cell-free DNA identifies KRAS copy number gain and mutation as a novel prognostic marker in Pancreatic cancer. Sci. Rep..

[29] Lemberger M., Loewenstein S., Lubezky N., Nizri E., Pasmanik-Chor M., Barazovsky E., Klausner J.M., Lahat G. (2019). MicroRNA profiling of pancreatic ductal adenocarcinoma (PDAC) reveals signature expression related to lymph node metastasis. Oncotarget.

[30] Vila-Navarro E., Vila-Casadesús M., Moreira L., Duran-Sanchon S., Sinha R., Ginés À., Fernández-Esparrach G., Miquel R., Cuatrecasas M., Castells A. (2016). MicroRNAs for Detection of Pancreatic Neoplasia. Ann. Surg..

[31] Aita A., Millino C., Sperti C., Pacchioni B., Plebani M., Pittà C.D., Basso D. (2021). Serum miRNA Profiling for Early PDAC Diagnosis and Prognosis: A Retrospective Study. Biomedicines.

[32] Dittmar R.L., Liu S., Tai M.C., Rajapakshe K., Huang Y., Longton G., DeCapite C., Hurd M.W., Paris P.L., Kirkwood K.S. (2021). Plasma miRNA Biomarkers in Limited Volume Samples for Detection of Early-stage Pancreatic Cancer. Cancer Prev. Res..

[33] Selleck M.J., Senthil M., Wall N.R. (2017). Making Meaningful Clinical Use of Biomarkers. Biomark. Insights.

[34] Hartwig W., Strobel O., Hinz U., Fritz S., Hackert T., Roth C., Büchler M.W., Werner J. (2013). CA19-9 in Potentially Resectable Pancreatic Cancer: Perspective to Adjust Surgical and Perioperative Therapy. Ann. Surg. Oncol..

[35] Kim S., Park B.K., Seo J.H., Choi J., Choi J.W., Lee C.K., Chung J.B., Park Y., Kim D.W. (2020). Carbohydrate antigen 19-9 elevation without evidence of malignant or pancreatobiliary diseases. Sci. Rep..

[36] Ferrone C.R., Finkelstein D.M., Thayer S.P., Muzikansky A., Fernandez-delCastillo C., Warshaw A.L. (2006). Perioperative CA19-9 levels can predict stage and survival in patients with resectable pancreatic adenocarcinoma. J. Clin. Oncol. Off. J. Am. Soc. Clin. Oncol..

[37] National Comprehensive Cancer Network (2022). Clinical Practice Guidelines in Oncology-Pancreatic Adenocarcinoma.

[38] Ballehaninna U.K., Chamberlain R.S. (2012). The clinical utility of serum CA 19-9 in the diagnosis, prognosis and management of pancreatic adenocarcinoma: An evidence based appraisal. J. Gastrointest. Oncol..

[39] Salomaa V., Pankow J., Heiss G., Cakir B., Eckfeldt J.H., Ellison R.C., Myers R.H., Hiller K.M., Brantley K.R., Morris T.L. (2000). Genetic background of Lewis negative blood group phenotype and its association with atherosclerotic disease in the NHLBI Family Heart Study. J. Intern. Med..

[40] Kim J.E., Lee K.T., Lee J.K., Paik S.W., Rhee J.C., Choi K.W. (2004). Clinical usefulness of carbohydrate antigen 19-9 as a screening test for pancreatic cancer in an asymptomatic population. J. Gastroenterol. Hepatol..

[41] Bedi M.M., Gandhi M.D., Jacob G., Lekha V., Venugopal A., Ramesh H. (2009). CA 19-9 to differentiate benign and malignant masses in chronic pancreatitis: Is there any benefit?. Indian J. Gastroenterol..

[42] Wu Z., Kuntz A.I., Wadleigh R.G. (2013). CA 19-9 tumor marker: Is it reliable? A case report in a patient with pancreatic cancer. Clin. Adv. Hematol. Oncol..

[43] Swords D.S., Firpo M.A., Scaife C.L., Mulvihill S.J. (2016). Biomarkers in pancreatic adenocarcinoma: Current perspectives. OncoTargets Ther..

[44] Hall C., Clarke L., Pal A., Buchwald P., Eglinton T., Wakeman C., Frizelle F. (2019). A Review of the Role of Carcinoembryonic Antigen in Clinical Practice. Ann. Coloproctol..

[45] Goonetilleke K.S., Siriwardena A.K. (2007). Systematic review of carbohydrate antigen (CA 19-9) as a biochemical marker in the diagnosis of pancreatic cancer. Eur. J. Surg. Oncol. (EJSO).

[46] Suzuki S., Shimoda M., Shimazaki J., Maruyama T., Oshiro Y., Nishida K., Sahara Y., Nagakawa Y., Tsuchida A. (2018). Predictive Early Recurrence Factors of Preoperative Clinicophysiological Findings in Pancreatic Cancer. Eur. Surg. Res..

[47] Scholler N., Urban N. (2007). CA125 in ovarian cancer. Biomark. Med..

[48] Jiang K., Tan E., Sayegh Z., Centeno B., Malafa M., Coppola D. (2017). Cancer Antigen 125 (CA125, MUC16) Protein Expression in the Diagnosis and Progression of Pancreatic Ductal Adenocarcinoma. Appl. Immunohistochem. Mol. Morphol..

[49] Luo G., Xiao Z., Long J., Liu Z., Liu L., Liu C., Xu J., Ni Q., Yu X. (2013). CA125 is Superior to CA19-9 in Predicting the Resectability of Pancreatic Cancer. J. Gastrointest. Surg..

[50] Chen T., Zhang M.-G., Xu H.-X., Wang W.-Q., Liu L., Yu X.-J. (2015). Preoperative Serum CA125 Levels Predict the Prognosis in Hyperbilirubinemia Patients With Resectable Pancreatic Ductal Adenocarcinoma. Medicine.

[51] Waters A.M., Der C.J. (2018). KRAS: The Critical Driver and Therapeutic Target for Pancreatic Cancer. Cold Spring Harb. Perspect. Med..

[52] Buscail L., Bournet B., Cordelier P. (2020). Role of oncogenic KRAS in the diagnosis, prognosis and treatment of pancreatic cancer. Nat. Rev. Gastroenterol. Hepatol..

[53] Downward J. (2003). Targeting RAS signalling pathways in cancer therapy. Nat. Rev. Cancer.

[54] Cheng F., Su L., Qian C. (2016). Circulating tumor DNA: A promising biomarker in the liquid biopsy of cancer. Oncotarget.

[55] Zou H.L., Wang Y., Gang Q., Zhang Y., Sun Y. (2017). Plasma level of miR-93 is associated with higher risk to develop type 2 diabetic retinopathy. Graefes Arch. Clin. Exp. Ophthalmol..

[56] Vila-Navarro E., Duran-Sanchon S., Vila-Casadesús M., Moreira L., Ginès À., Cuatrecasas M., Lozano J.J., Bujanda L., Castells A., Gironella M. (2019). Novel Circulating miRNA Signatures for Early Detection of Pancreatic Neoplasia. Clin. Transl. Gastroenterol..

[57] Chen Y., Li Z., Zhang M., Wang B., Ye J., Zhang Y., Tang D., Ma D., Jin W., Li X. (2019). Circ-ASH2L promotes tumor progression by sponging miR-34a to regulate Notch1 in pancreatic ductal adenocarcinoma. J. Exp. Clin. Cancer Res..

[58] Deng S., Wang J., Zhang L., Li J., Jin Y. (2021). LncRNA HOTAIR Promotes Cancer Stem-Like Cells Properties by Sponging miR-34a to Activate the JAK2/STAT3 Pathway in Pancreatic Ductal Adenocarcinoma. OncoTargets Ther..

[59] Yao L.-C., Jiang X.-H., Yan S.-S., Wang W., Wu L., Zhai L.-L., Xiang F., Ji T., Ye L., Tang Z.-G. (2021). Four potential microRNAs affect the progression of pancreatic ductal adenocarcinoma by targeting MET via the PI3K/AKT signaling pathway. Oncol. Lett..

[60] Brabletz T. (2012). EMT and MET in Metastasis: Where Are the Cancer Stem Cells?. Cancer Cell.

[61] Li Z., Tao Y., Wang X., Jiang P., Li J., Peng M., Zhang X., Chen K., Liu H., Zhen P. (2018). Tumor-Secreted Exosomal miR-222 Promotes Tumor Progression via Regulating P27 Expression and Re-Localization in Pancreatic Cancer. Cell. Physiol. Biochem..

[62] Sharma S.S., Pledger W.J. (2016). The non-canonical functions of p27(Kip1) in normal and tumor biology. Cell Cycle.

[63] Tai Y.-L., Chen K.-C., Hsieh J.-T., Shen T.-L. (2018). Exosomes in cancer development and clinical applications. Cancer Sci..

[64] Doyle L.M., Wang M.Z. (2019). Overview of Extracellular Vesicles, Their Origin, Composition, Purpose, and Methods for Exosome Isolation and Analysis. Cells.

[65] McAndrews K.M., Kalluri R. (2019). Mechanisms associated with biogenesis of exosomes in cancer. Mol. Cancer.

[66] Bor R., Madácsy L., Fábián A., Szepes A., Szepes Z. (2015). Endoscopic retrograde pancreatography: When should we do it?. World J. Gastrointest. Endosc..

[67] Meng M., Zhong K., Jiang T., Liu Z., Kwan H.Y., Su T. (2021). The current understanding on the impact of KRAS on colorectal cancer. Biomed. Pharmacother..

[68] Westcott P.M.K., To M.D. (2013). The genetics and biology of KRAS in lung cancer. Chin. J. Cancer.

[69] Principe D.R., Underwood P.W., Korc M., Trevino J.G., Munshi H.G., Rana A. (2021). The Current Treatment Paradigm for Pancreatic Ductal Adenocarcinoma and Barriers to Therapeutic Efficacy. Front. Oncol..

[70] Gall T.M., Jacob J., Frampton A.E., Krell J., Kyriakides C., Castellano L., Stebbing J., Jiao L.R. (2014). Reduced dissemination of circulating tumor cells with no-touch isolation surgical technique in patients with pancreatic cancer. JAMA Surg..

[71] Yoshimasu T., Maebeya S., Suzuma T., Bessho T., Tanino H., Arimoto J., Sakurai T., Naito Y. (1999). Disappearance Curves for Tumor Markers after Resection of Intrathoracic Malignancies. Int. J. Biol. Markers.

[72] Diaz L.A., Bardelli A. (2014). Liquid biopsies: Genotyping circulating tumor DNA. J. Clin. Oncol..

[73] Pai R.K., Beck A.H., Mitchem J., Linehan D.C., Chang D.T., Norton J.A. (2011). Pattern of lymph node involvement and prognosis in pancreatic adenocarcinoma: Direct lymph node invasion has similar survival to node-negative disease. Am. J. Surg. Pathol..

[74] Mikamori M., Yamada D., Eguchi H., Hasegawa S., Kishimoto T., Tomimaru Y., Asaoka T., Noda T., Wada H., Kawamoto K. (2017). MicroRNA-155 Controls Exosome Synthesis and Promotes Gemcitabine Resistance in Pancreatic Ductal Adenocarcinoma. Sci. Rep..

[75] Kong X., Du Y., Wang G., Gao J., Gong Y., Li L., Zhang Z., Zhu J., Jing Q., Qin Y. (2011). Detection of differentially expressed microRNAs in serum of pancreatic ductal adenocarcinoma patients: miR-196a could be a potential marker for poor prognosis. Dig. Dis. Sci..

[76] Thiery J.P. (2002). Epithelial-mesenchymal transitions in tumour progression. Nat. Rev. Cancer.

[77] Wang Y.-N., Chen Z.-H., Chen W.-C. (2017). Novel circulating microRNAs expression profile in colon cancer: A pilot study. Eur. J. Med. Res..

[78] Yang X., Yan Z., Yang H., Ni H., Zhang L., Wang Y. (2019). Clinical value of combined detection of miR-1202 and miR-195 in early diagnosis of cervical cancer. Oncol. Lett..

[79] Quan Y., Song Q., Wang J., Zhao L., Lv J., Gong S. (2017). MiR-1202 functions as a tumor suppressor in glioma cells by targeting Rab1A. Tumor Biol..

[80] Wang C., Yoo Y., Fan H., Kim E., Guan K.-L., Guan J.-L. (2010). Regulation of Integrin β1 Recycling to Lipid Rafts by Rab1a to Promote Cell Migration*. J. Biol. Chem..

[81] Yang Z., LaRiviere M.J., Ko J., Till J.E., Christensen T., Yee S.S., Black T.A., Tien K., Lin A., Shen H. (2020). A Multianalyte Panel Consisting of Extracellular Vesicle miRNAs and mRNAs, cfDNA, and CA19-9 Shows Utility for Diagnosis and Staging of Pancreatic Ductal Adenocarcinoma. Clin. Cancer Res..

[82] Weng Y.R., Cui Y., Fang J.Y. (2012). Biological functions of cytokeratin 18 in cancer. Mol. Cancer Res..

[83] Khushman M., Patel G.K., Laurini J.A., Bhardwaj A., Roveda K., Donnell R., Sherling K., Case B., Frankel A.E., Pai S. (2019). Exosomal markers (CD63 and CD9) expression and their prognostic significance using immunohistochemistry in patients with pancreatic ductal adenocarcinoma. J. Gastrointest. Oncol..

[84] Liu M., Xu A., Yuan X., Zhang Q., Fang T., Wang W., Li C. (2015). Downregulation of microRNA-409-3p promotes aggressiveness and metastasis in colorectal cancer: An indication for personalized medicine. J. Transl. Med..

[85] Song Q., Ji Q., Xiao J., Li F., Wang L., Chen Y., Xu Y., Jiao S. (2018). miR-409 Inhibits Human Non-Small-Cell Lung Cancer Progression by Directly Targeting SPIN1. Mol. Ther. Nucleic Acids.

[86] Wu L., Zhang Y., Huang Z., Gu H., Zhou K., Yin X., Xu J. (2019). MiR-409-3p Inhibits Cell Proliferation and Invasion of Osteosarcoma by Targeting Zinc-Finger E-Box-Binding Homeobox-1. Front. Pharmacol..

[87] Zhang F.B., Du Y., Tian Y., Ji Z.G., Yang P.Q. (2019). MiR-1299 functions as a tumor suppressor to inhibit the proliferation and metastasis of prostate cancer by targeting NEK2. Eur. Rev. Med. Pharmacol. Sci..

[88] Vicentini C., Calore F., Nigita G., Fadda P., Simbolo M., Sperandio N., Luchini C., Lawlor R.T., Croce C.M., Corbo V. (2020). Exosomal miRNA signatures of pancreatic lesions. BMC Gastroenterol..

[89] Butera G., Pacchiana R., Mullappilly N., Margiotta M., Bruno S., Conti P., Riganti C., Donadelli M. (2018). Mutant p53 prevents GAPDH nuclear translocation in pancreatic cancer cells favoring glycolysis and 2-deoxyglucose sensitivity. Biochim. Biophys. Acta (BBA) Mol. Cell Res..

